# Voice Multilateration System

**DOI:** 10.3390/s21113890

**Published:** 2021-06-04

**Authors:** Robert Burczyk, Krzysztof Cwalina, Malgorzata Gajewska, Jaroslaw Magiera, Piotr Rajchowski, Jaroslaw Sadowski, Jacek Stefanski

**Affiliations:** Faculty of Electronics, Telecommunications and Informatics, Gdansk University of Technology, 80-233 Gdansk, Poland; robert.burczyk@pg.edu.pl (R.B.); krzysztof.cwalina@eti.pg.edu.pl (K.C.); malgorzata.gajewska@eti.pg.edu.pl (M.G.); jaroslaw.magiera@eti.pg.edu.pl (J.M.); piotr.rajchowski@eti.pg.edu.pl (P.R.); jacek.stefanski@eti.pg.edu.pl (J.S.)

**Keywords:** radio navigation, wireless sensor networks, aircraft navigation, MLAT, VCS

## Abstract

This paper presents an innovative method of locating airplanes, which uses only voice communication between an air traffic controller and the pilot of an aircraft. The proposed method is described in detail along with its practical implementation in the form of a technology demonstrator (proof of concept), included in the voice communication system (VCS). A complete analysis of the performance of the developed method is presented, including the results of simulation and measurement tests in real conditions. The obtained results are very optimistic and indicate that the proposed solution may constitute an alternative method of locating aircraft in emergency conditions, i.e., a backup solution in the case of failure of other positioning systems.

## 1. Introduction

Currently, many systems providing the position of the aircraft in various phases of its flight are used in the world. The aviation radio navigation systems in use can be divided into two main groups: passive systems, in which the aircraft relies solely on the received information in the navigation process, and active systems, in which the aircraft participates both in receiving and transmitting navigation information. The first group of systems includes mainly [1]:

- Instrument landing system (ILS);
- Microwave landing system (MLS);
- VHF omnidirectional range (VOR) and Doppler VHF omnidirectional range (DVOR);
- Non-directional beacons–automatic direction finder (NDB–ADF);
- Global positioning system (GPS).

In turn, the second group of aviation radio navigation systems includes mainly:

- Distance measuring equipment (DME);
- Radio altimeter (RA);
- Wide area multilateration (WAM).

The above-mentioned systems are recommended by the international civil aviation organization (ICAO), which is also an organization standardizing individual solutions.

From the systems mentioned above, the greatest attention is now paid to development and implementation of multilateration (MLAT) or wide area multilateration (WAM) systems which are considered as low-cost alternatives to radar-based aircraft positioning [2,3,4]. The MLAT system consists of many measurement sensors deployed in selected places with known coordinates in a given supervision area. Synchronized ground receivers measure the time of arrival of signals radiated from transmitters onboard aircraft. The position of the aircraft is then estimated using the time difference of arrival (TDoA) method [5]. Various types of signals can be used to estimate the position of the aircraft. These can be signals transmitted by the on-board transponder in response to signals from the secondary surveillance radar (SSR), automatic dependent surveillance–broadcast (ADS–B) signals, or DME signals [6,7]. We know how important it is to know the current position of the aircraft in each phase of its flight. Therefore, it is necessary to develop systems that will ensure the tracking of the aircraft position in all conditions. It is commonly believed that information about the position of an airplane should be provided to the air control center (ACC) from many (at least two) independent sources and any possibility to estimate aircraft position using another, independent data source should be taken into account. Therefore, authors decided to investigate the possibility to track aircraft using a very high frequency (VHF) voice communication of aircraft pilots and an air traffic control (ATC) using the MLAT principle. Such narrowband analog transmissions will probably not provide good quality of positioning due to irregular waveform shape and a short duration time of transmissions from the transmitters onboard aircraft [2]. However, as no reliable data on possible accuracy of positioning using VHF AM signals could be found in the literature, authors implemented a positioning subsystem as part of the development of a voice communication system (VCS) for ground to air ATC communication.

According to this idea, as an alternative solution, an aircraft location system was proposed using audio signals that are available during the operation of the VCS [8,9,10]. The VCS system provides robust voice communication between an air traffic controller and aircraft pilots. These signals were the basis for the development and construction of a prototype MLAT system, which uses speech signals for aircraft position estimation (VCS-MLAT) [11].

Analog voice communication is not a perfect solution for aircraft tracking due to the following disadvantages. The first one is the variability of the transmitted waveforms. The transmitted messages and the voice characteristics of speaking people varies; therefore, the quality of the TDoA measurements based on the correlation of the audio signals recorded by the MLAT ground stations will also differ from one transmission to another. The second disadvantage is an impossibility of automatic identification of a transmitting station. Therefore, any solution based on analog voice signals will only estimate the position of aircraft, without the identification. However, as the majority of the ATC communication is still conducted using the analog VHF AM transceivers (backward compatibility especially for emergency communication), the proposed positioning subsystem VCS-MLAT may still provide useful data, especially in the case of failure or intentional jamming of primary positioning and tracking systems.

MLAT systems enable the tracking of not only aircraft but also airport vehicles within the airport. The main documents standardizing the work of MLAT systems are the publications of the European Organization for Civil Aviation Equipment (EUROCAE) [12,13]. These technical specifications set out minimum performance requirements for those systems that provide air traffic controllers with real-time information on the airspace situation. The results of the practical verification of aircraft location accuracy in MLAT systems available in the literature concern solutions in which the source of signals for distributed reference stations are SSR and ADS–B systems [14,15,16]. However, the synchronization of individual reference stations is performed using GPS receivers [17,18]. An interesting solution, proven in simulation studies, is the asynchronous WAM system [19], in which individual reference stations work asynchronously with each other and the process of locating the aircraft is based on changing the position of the aircraft in two or more moments.

The requirements for aircraft positioning accuracy depend on the phase of the flight [20]. In a typical implementation of the MLAT system, which uses pulse-based transmission (secondary surveillance radar or ADS–B transponders), the error in determining the horizontal position of the aircraft does not exceed 350 m along the route and 150 m in the airport area; such high accuracy is available only by using wide bandwidth of transmitted signals, much wider than in VHF AM communication. Therefore, a direct comparison of results presented in the literature for standard MLAT implementations and results from our investigation is not possible.

This paper presents the results of simulation and measurement tests of an innovative method of locating aircraft based on the received audio signals in the aviation frequency band in the range of 118–136 MHz. The proposed system may be a backup solution in case of failure of other positioning or surveillance systems. In extreme cases, the only source of information for ATC is voice communication with the pilot, which, as we will see later in the paper, can be used to estimate the position of the aircraft during transmission. Such supplementary positional data may be invaluable when aircraft pilot reports are incorrect or inaccurate. Another possible application may be quick verification of transmitting aircraft in case of absence of the aircraft callsign in transmitted message.

This paper is organized as follows: Section 2 describes the proposed solution. In Section 3 the simulation studies are presented. Section 5 is divided into four subsections, which present step-by-step digital signal processing procedures applied during the practical verification of the proposed location method. Finally, the last section concludes the paper.

## 2. Description of the Proposed Method

**Figure 1:**
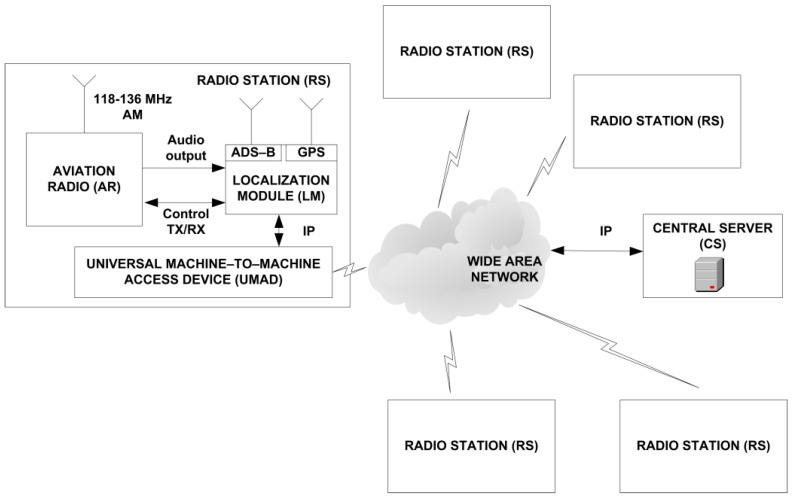
Technology demonstrator for testing the new method of locating aircraft in the VCS system.

As part of the research and development work carried out, a new functionality was implemented in the currently developed VCS system, which as it is known supports voice communication in civil and military air traffic, both analog in the ground-to-ground and ground-to-air connection. The standardization documents of the VCS system, developed as part of the EUROCAE organization, do not provide such functionality of locating aircraft on the basis of audio signals from aviation radio stations located in the monitored area [8,9,10]. Therefore, a new functionality was introduced to the VCS without changing the system assumptions. The VCS system specification uses IP protocol and IP-based links between all components. In addition to upper layers protocols defined by VCS standardization body, it was possible to add new functionalities with custom protocols using the same links and communication network maintaining backward system compatibility. Figure 1 shows the architecture of a technology demonstrator for the practical verification of the innovative method of locating aircraft based on the received audio signals in the air frequency band in the proposed VCS system.

Five radio stations (RS) were developed and manufactured, which worked in a distributed VCS system. Each radio station consists of the following components:

- Aviation radio (AR) operating in the 118 MHz–136 MHz band with a control interface, acts as a receiver of audio signals with amplitude modulation (AM);
- Universal machine-to-machine access device (UMAD)—3G/4G router and gateway providing RS connection with VCS network;
- Localization module (LM), responsible for data acquisition from the AR receiver and their initial preprocessing;
- Central server (CS), responsible for the aircraft position estimation process;
- GPS receiver, necessary for synchronizing data sent to the central server (timestamp);
- ADS–B signal receiver, providing information about the actual position of the aircraft to the central server CS for comparison purposes (reference data for evaluation of position estimation quality).

**Figure 2:**
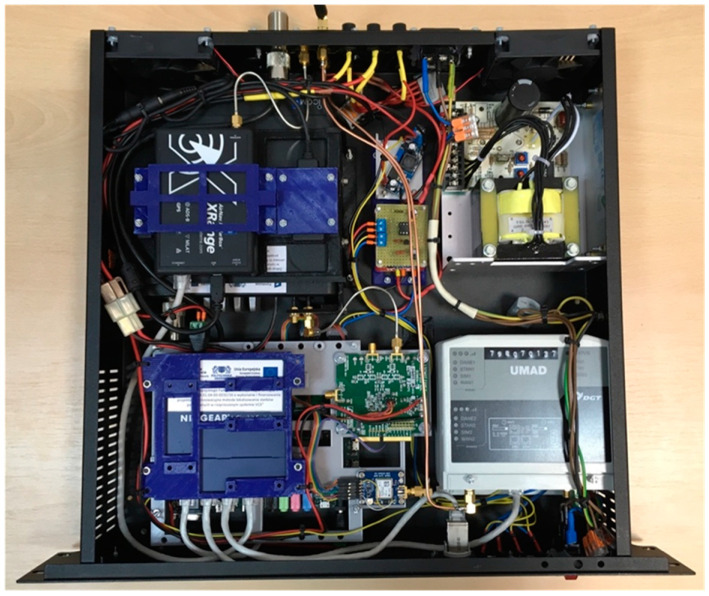
Interior of the radio station (RS).

Each of the radio stations was assembled in a housing that meets the 19-inch standard. Outside the housing, only the antennas of aviation radio, ADS–B, and GPS receivers were led. Figure 2 shows the assembled RS. General purpose commercial off the shelf components and modules were used to build the RS, while the uniqueness of the solution was determined by the software based on an evaluation board with a system on a chip (SoC) technology, consisting of an advanced RISC machine (ARM) processor and a field programmable gate array (FPGA) matrix.

The innovation of the demonstrator solution consisted, on one hand, of building a localization module (LM), and, on the other hand, of developing a method (and then an algorithm and its implementation on a dedicated hardware solution) for the purpose of estimating the position of aircraft based on speech signals received by 5 radio stations (RS). For synchronization purposes, each ML is equipped with a GPS receiver.

**Figure 3:**
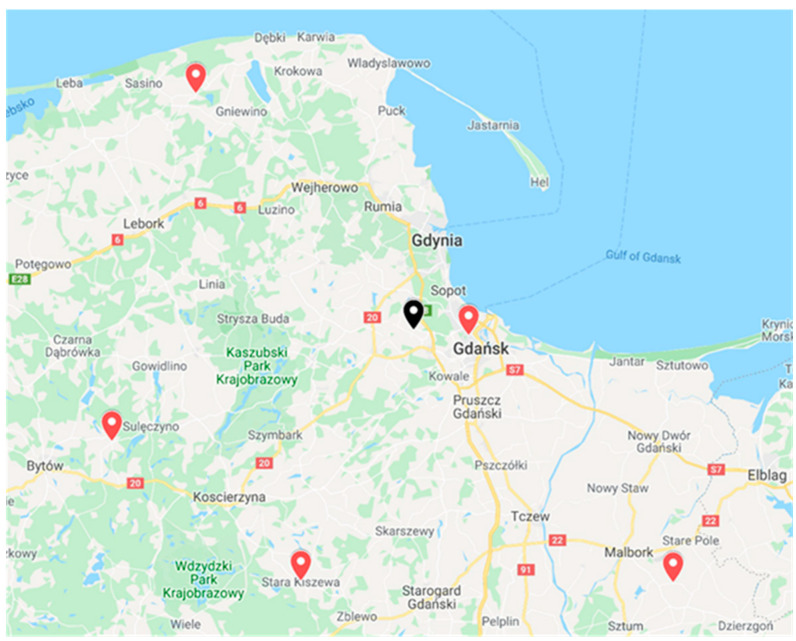
Map with marked locations of radio stations (RS) (map background from Google Maps).

During tests in a real environment, the radio stations were deployed in the northern part of Poland, in the Pomeranian Voivodeship (Szropy, Choczewo, Stara Kiszewa, Parchowo, and Gdansk). Figure 3 shows the map along with the radio station installation locations marked in red, and the nearest international airport, i.e., Gdansk Lech Walesa Airport. Radio stations are located approximately 60 km from the airport, with the exception of the nearest RS located on the building of the Faculty of Electronics, Telecommunications and Informatics of the Gdansk University of Technology.

First, a methodology was developed to collect reliable data for the purpose of aircraft location estimation. The tests carried out concerned several stages from the analysis of voice data to the determination of the position of the flying object and saving it in the database on the central server (CS) of the VCS system.

The first step in the aircraft positioning process is selecting a set of recorded signals for the same audio transmission received by different radio stations (RS). The grouping of signals is based on the comparison of the timestamps of their recording start (these markers are saved in the database). The set includes signals that markers differ by no more than a preset time interval. This interval is defined by the operator and is one of the configuration parameters of the program for determining the time difference TDoA. The value of the maximum interval between the timestamps of the audible signals was set to 500 ms.

In the second stage, the differences in propagation times TDoA of radio signals originating from the onboard transmitter and received by all pairs of radio stations were determined. From the tests carried out with the use of the technology demonstrator and after implementing many additional functions in the algorithm itself, taking into account, e.g., filtering eliminating interference outside the band of speech signals, detection and elimination of the initial and final transmission interval containing noise related to switching on and off the transmission, and corrections to the function of calculating the correlation of signals, the standard deviation of TDoA times was obtained at the level of 1 μs and the difference between the maximum and minimum TDoA value did not exceed 5 μs. These are the values to be expected with the good quality transmission of signals received by radio stations.

In the last stage, the aircraft location was estimated. To determine the accuracy of this estimation, the calculated position was compared with the reference data reported in the ADS–B system messages.

Next, this paper will present important implementation details, the results of simulation tests of the potential effectiveness of the localization system operation, and the results of experimental studies with the use of the technology demonstrator.

## 3. Aircraft Position Estimation Algorithms

In the VCS system, the position of the aircraft is determined based on the measurement of the time difference of arrival TDoA radio signals to the radio stations (reference stations) located at predetermined locations. These stations receive signals transmitted by airborne radio transceivers, operating in VHF band. The synchronous reception and central processing of these signals enable the TDoA value to be determined.

The relationship between the unknown aircraft position and the TDoA values and RS positions is described by the system of positional equations:

(1) $$\begin{array}{l} r_{i,j}=c\cdot \Delta T_{i,j}=d_{i,j}+\varepsilon_{i,j}=\left\|x-x_{i}\right\|-\left\|x-x_{j}\right\|+\varepsilon_{i,j}\\ =\sqrt{{\left(x-x_{i}\right)}^{2}+{\left(y-y_{i}\right)}^{2}+{\left(z-z_{i}\right)}^{2}}\\ -\sqrt{{\left(x-x_{j}\right)}^{2}+{\left(y-y_{j}\right)}^{2}+{\left(z-z_{j}\right)}^{2}}+\varepsilon_{i,j} \end{array}$$

where **x** = [*x*,*y*,*z*]^T^ is a vector of the unknown coordinates of the aircraft position, **x***_i_* = [*x_i_*,*y_i_*,*z_i_*]^T^ are the coordinates of the position of the *i*-th reference station, and Δ*T_i,j_* is the TDoA value relative to the *i*-th and *j*-th reference station. The symbol *r_i,j_* denotes the estimated difference in the distance of the object from the station *i* and *j*, *d_i,j_* the real difference of this distance; *ε_i,j_* is the error in estimating this difference. The symbol *c* denotes the speed of propagation of the electromagnetic wave.

The station with index *j* is the reference point for determining TDoA, common to all measurements. Thus, when *N* reference stations are used, a system of *N* − 1 positional equations is obtained. In the two-dimensional case, they represent the hyperbole equations, and in the three-dimensional case, they represent the hyperboloid. To determine the position of an object on a plane, it is necessary to use at least three stations, and for a three-dimensional position, it is necessary to use four. However, in this case, there is a possibility of solution ambiguity (two possible solutions). To ensure an unambiguous solution, it is necessary to increase the number of reference stations by one.

Solving the TDoA positional equation system is problematic for two reasons. First, the equations are nonlinear, which makes it difficult to obtain a solution by conventional methods used in linear systems. Second, the error values *ε_i,j_* for different pairs of reference stations are mutually independent, which leads to a contradiction in the system of equations. Therefore, position estimation algorithms serve to determine the approximate position best suited to the measured parameter values.

Localization algorithms can be classified according to the course of the procedure. Some algorithms have so-called closed form, which means that the position is obtained after one run of the algorithm (set sequence of steps). The second group consists of iterative algorithms, for which initialization is required to provide an estimated solution. In iterative algorithms, some steps are repeated (in a loop) to obtain a better and better-suited solution, until an acceptably small error is obtained or a specified number of iterations are performed.

After the literature review, two localization algorithms were selected that can be used in the discussed system. These are the spherical interpolation (SI) [21] algorithms belonging to the first group and the Foy iterative algorithm [22]. It is worth mentioning here that the algorithms used to estimate the position of the aircraft were not modified but only adapted to work with samples of voice signals. The choice of these two algorithms was dictated by their widespread use in positioning systems.

### 3.1. Spherical Interpolation Algorithm

The description of algorithms requires a certain convention. The position of the object is assumed to be in the local Cartesian coordinate system. The origin of this system is at the location of the reference station with index 1, which is the reference point for TDoA measurements. The symbol *R* is used to denote the distance of the searched object from the beginning of the coordinate system, i.e., *R* = ‖**x**‖. Similarly, *R_i_* denotes the distance of the *i*-th reference station from the origin of the coordinate system, i.e., *R_i_* = ‖**x***_i_*‖.

Assuming the above, the system of positional equations can be written as follows

(2) $$\varepsilon_{i,1}=R_{i}^{2}-d_{i,1}^{2}-2Rd_{i,1}-2x_{i}^{T}x\;i=2,\;\ldots ,N$$

This system can be written in a matrix form

(3) ε=δ−2Rd−2Sx

where

$$\varepsilon =\left[\begin{array}{c} \varepsilon_{2,1}\\ \varepsilon_{3,1}\\ \vdots \\ \varepsilon_{N,1} \end{array}\right]\;\delta =\left[\begin{array}{c} R_{2}^{2}-d_{2,1}^{2}\\ R_{3}^{2}-d_{3,1}^{2}\\ \vdots \\ R_{N}^{2}-d_{N,1}^{2} \end{array}\right]\;d=\left[\begin{array}{c} d_{2,1}\\ d_{3,1}\\ \vdots \\ d_{N,1} \end{array}\right]\;S=\left[\begin{array}{ccc} x_{2} & y_{2} & z_{2}\\ x_{3} & y_{3} & z_{3}\\ \vdots & \vdots & \vdots \\ x_{N} & y_{N} & z_{N} \end{array}\right]$$

The solution of this system of equations using the least squares method, minimizing the mean square error ***ε***^T^***ε***, can be written as follows

(4) $$x=\frac{1}{2}S_{W}^{*}\left(\delta -2Rd\right)$$

where

$$S_{W}^{*}={\left(S^{T}S\right)}^{-1}S^{T}$$

The SI algorithm is based on substituting Equation (4) to Equation (3) and determining the *R* value that minimizes the estimation error. After substitution, a relationship is obtained

(5) $$\hat{\varepsilon }=\delta -2Rd-{\mathrm{SS}}_{W}^{*}\left(\delta -2Rd\right)=\left(I-{\mathrm{SS}}_{W}^{*}\right)\left(\delta -2Rd\right)$$

where **I** is the identity matrix of size (*N* − 1)∙(*N* − 1).

Defining auxiliary matrices

$$\begin{array}{l} P_{S}={\mathrm{SS}}_{W}^{*}=S{\left(S^{T}S\right)}^{-1}S^{T}\\ P_{S}^{⊥}=I-P_{S} \end{array}$$

Equation (5) can be written as

(6) $$\hat{\varepsilon }=P_{S}^{⊥}\left(\delta -2Rd\right)$$

In this case, the object distance from the reference station is estimated as follows

(7) $$R=\frac{d^{T}P_{S}^{⊥}P_{S}^{⊥}\delta }{2d^{T}P_{S}^{⊥}P_{S}^{⊥}d}$$

After substituting (7) to (4), the formula for estimating the position of the object is obtained

(8) $$\hat{x}=\frac{1}{2}{\left(S^{T}S\right)}^{-1}S^{T}\left(I-\frac{{\mathrm{dd}}^{T}P_{S}^{⊥}P_{S}^{⊥}}{2d^{T}P_{S}^{⊥}P_{S}^{⊥}d}\right)\delta$$

### 3.2. Foy Algorithm

The Foy algorithm is an iterative algorithm in which to solve a system of nonlinear equations; their approximation is used by the first two components of expanding these equations into the Taylor series. Let (*x*^0^,*y*^0^,*z*^0^) denote the coordinates of the real position of the airplane and (*x_i_*,*y_i_*,*z_i_*) represent the real coordinates of the known position of the *i*-th reference station. Then the following relationship can be written

(9) $$f_{k}\left(x^{0},y^{0},z^{0},x_{i},y_{i},z_{i}\right)=m_{ik}-e_{k}k=1,\;2,\;\ldots ,\;n$$

where:

- *f_k_*(*x*^0^,*y*^0^,*z*^0^,*x_i_*,*y_i_*,*z_i_*) represents the actual values of the measured quantities;
- *m_ik_*—value of the measured quantity;
- *e_k_*—error of *m_ik_* measurement;
- *n*—number of measurements made between the airplane and the given reference station.

The mathematical problem here is to find the value (*x*^0^,*y*^0^,*z*^0^) if there are n measurement results *m_ik_* and the form of the function *f_k_*(·). During calculations, it is assumed that the expected value of errors E[*e_k_*] = 0. However, the element *kj* of the covariance matrix of measurement errors **Σ** = [*σ_kj_*] can be written as *σ_kj_* = E[*e_k_e_j_*]. If the coordinates of the estimated position are marked as (*x_v_*,*y_v_*,*z_v_*) then the coordinates of the real position can be written

(10) $$\begin{array}{l} x^{0}=x_{v}+\delta_{x}\\ y^{0}=y_{v}+\delta_{y}\\ z^{0}=z_{v}+\delta_{z} \end{array}$$

where *δ_x_*, *δ_y_*, and *δ_z_* represent errors in determining individual coordinates. Second-order Taylor series expansion of the function *f_k_* is as follows

(11) $$f_{kv}+a_{k1}\delta_{x}+a_{k2}\delta_{y}+a_{k3}\delta_{z}≅m_{ik}-e_{k}$$

where f_kv_ = f_k_(x_v_,y_v_,z_v_,x_i_,y_i_,z_i_),

$$\begin{array}{c} f_{kv}=f_{k}(x_{v},y_{v},z_{v},x_{i},y_{i},z_{i}),\\ a_{k1}={\frac{\partial f_{k}}{\partial x}}_{|\begin{array}{l} x=x_{v}\\ y=y_{v}\\ z=z_{v} \end{array}}a_{k2}={\frac{\partial f_{k}}{\partial y}}_{|\begin{array}{l} x=x_{v}\\ y=y_{v}\\ z=z_{v} \end{array}}a_{k3}={\frac{\partial f_{k}}{\partial z}}_{|\begin{array}{l} x=x_{v}\\ y=y_{v}\\ z=z_{v} \end{array}} \end{array}$$

For the purposes of the above equation, the following matrices and vectors can be defined

(12) $$A=\left[\begin{array}{ccc} a_{11} & a_{12} & a_{13}\\ a_{21} & a_{22} & a_{23}\\ \vdots & \vdots & \vdots \\ a_{n1} & a_{n2} & a_{n3} \end{array}\right]\;\delta =\left[\begin{array}{c} \delta_{x}\\ \delta_{y}\\ \delta_{z} \end{array}\right]\;z=\left[\begin{array}{c} m_{i1}-f_{1v}\\ m_{i2}-f_{2v}\\ \vdots \\ m_{in}-f_{nv} \end{array}\right]\;e=\left[\begin{array}{c} e_{1}\\ e_{2}\\ \vdots \\ e_{n} \end{array}\right]$$

Thus, Expression (11) can be written as follows

(13) Aδ≅z−e

In each iteration step, one should find **δ** that minimizes the sum of squared errors

(14) $$\delta ≅{\left[A^{T}\Sigma^{-1}A\right]}^{-1}A^{T}\Sigma^{-1}z$$

Then the estimated coordinates (*x_v_*,*y_v_*,*z_v_*) should be replaced with new values, respectively *x_v_* + *δ_x_*, *y_v_* + *δ_y_*, and *z_v_* + *δ_z_*. These values can be used in the next iteration step or be the result of the algorithm. The error covariance matrix of the position estimated in this way can be written with the relationship

(15) $$Q_{0}≅{\left[A^{T}\Sigma^{-1}A\right]}^{-1}$$

When the elements of the error vector **e** are mutually independent with the same variance *σ*^2^, instead of Formula (14) one can use (16) to determine the vector **δ**

(16) $$\delta ≅{\left[A^{T}A\right]}^{-1}A^{T}z$$

In this situation, the error covariance matrix of the estimated position will be as follows

(17) $$Q_{0}≅{\left[A^{T}A\right]}^{-1}\sigma^{2}$$

The above-mentioned relationships are a general presentation of the Foy algorithm. It is important to properly define the function *f_k_*. In the case of the considered radiolocation system, the input data of the algorithm will be measurements of differences in the time of arrival of radio signals to individual reference stations, which are then used to determine the location of the object.

## 4. Results of Simulation Tests

To compare the operation of the above-described aircraft position estimation algorithms, computer simulations were carried out in the MATLAB environment [23].

The simulation was carried out assuming the availability of five reference stations, located in places with geographic coordinates shown in Table 1, and they are consistent with the actual values of the stations in Figure 3.

**Figure 4:**
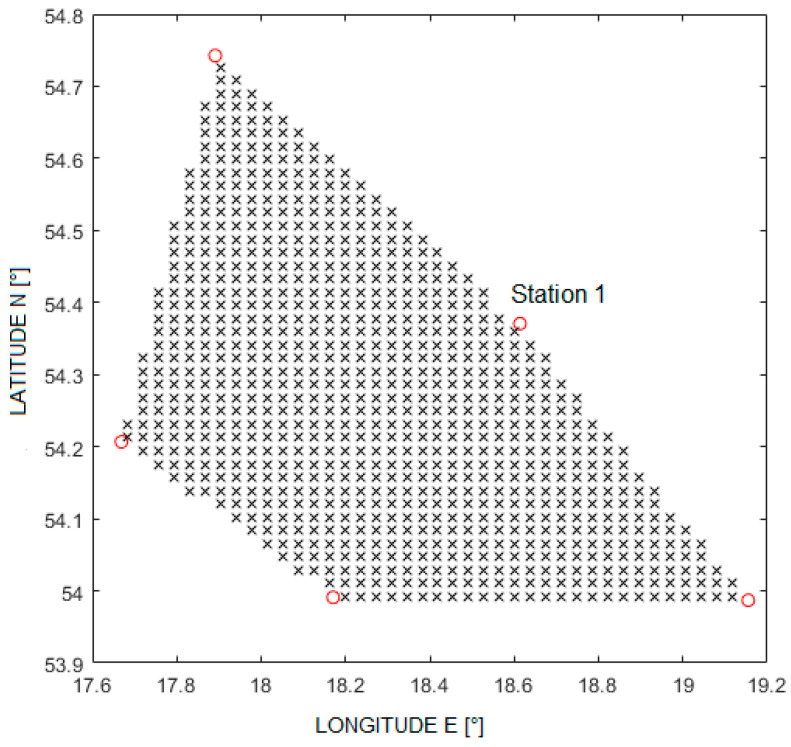
Arrangement of reference stations (‘o’ symbol) and analyzed positions (‘x’ symbol).

The positions of the object were analyzed inside the pentagon, the vertices of which define the positions of reference stations. To distinguish these positions, a 50 × 50 points grid was first generated with coordinates ranging from 53.9° to 54.8° latitude and 17.5° to 19.3° longitude. The height of all points has been set at 10,000 m above sea level. Then, from this mesh of 2500 points, only those inside the polygon were selected, resulting in a set of 850 positions. Their distribution is shown in Figure 4. It was decided to conduct research only inside the area limited by radio stations because, as it turned out during the experimental verification, transmissions originated from planes outside the area were rarely received by at least three ground stations or the signal-to-noise ratio was too low in distant stations to obtain reliable position indication.

For each of the analyzed positions, the actual TDoA values were determined. Station number 1 was taken as the reference station. To determine the distances from the station, it is necessary to transform the position from the geographic system to the Cartesian system, where the coordinates are expressed in meters. The east north up (ENU) system was adopted here, the origin of which coincides with the location of the first station, the XY plane is tangent to the Earth’s surface and the X axis is directed to the east.

To the actual measurements of TDoA, an error was added which was a random variable with a uniform distribution in the range from ±0.5 µs to ±5.0 µs. Then the localization algorithm function was called, to which the coordinates of the reference stations and the error-bearing TDoA values were transferred. One hundred iterations of the algorithm were performed for each aircraft position in the grid with randomly generated measurement errors, and then the root-mean-square (RMS) of estimated position errors was calculated as a measure of position estimation uncertainty.

The simulation research focused on determining the position of the object in the horizontal plane (2D) only. Typically, aircraft must maintain horizontal separation of at least several kilometers so horizontal position estimation with required accuracy is possible in our system, but the aircraft flight altitude must be known or maintained with accuracy reaching tens of meters while errors in altitude estimation, which we observed in our system, exceeded hundreds of meters. This made 3D positioning impractical. Therefore, during the simulation the true aircraft position (variable latitude and longitude, fixed altitude) was used to generate TDoA data but position estimation error evaluation was performed in 2D only by setting both true and estimated aircraft altitude to zero in WGS84 geographic system. After the conversion of WGS84 latitude/longitude to Cartesian coordinate system (earth centered, earth fixed—ECEF) position estimation errors were calculated as the square root of squared differences in x/y/z coordinates of zero-altitude true and estimated aircraft position.

**Figure 5:**
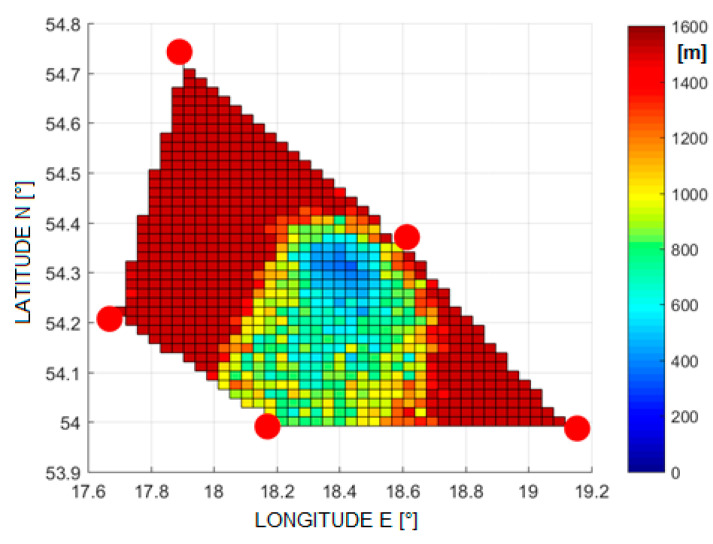
The RMSE of 2D position estimation in (m) for the SI algorithm (TDoA error is ±2 µs).

**Figure 6:**
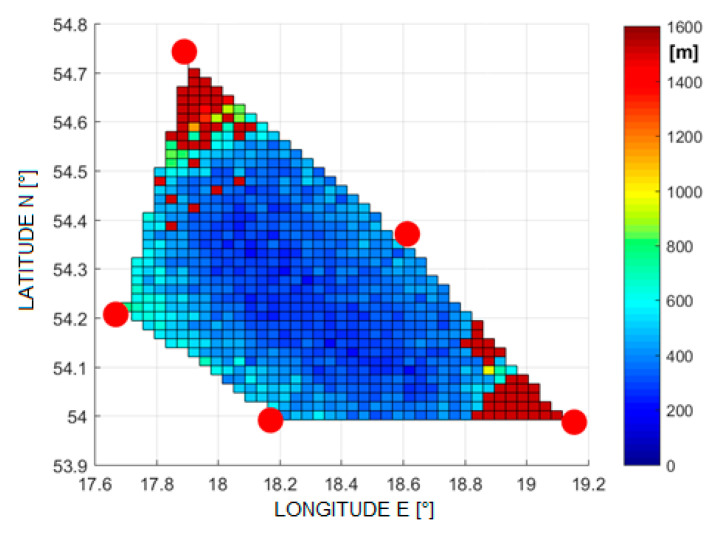
The RMSE of 2D position estimation in (m) for the Foy algorithm (TDoA error is ±2 µs).

**Figure 7:**
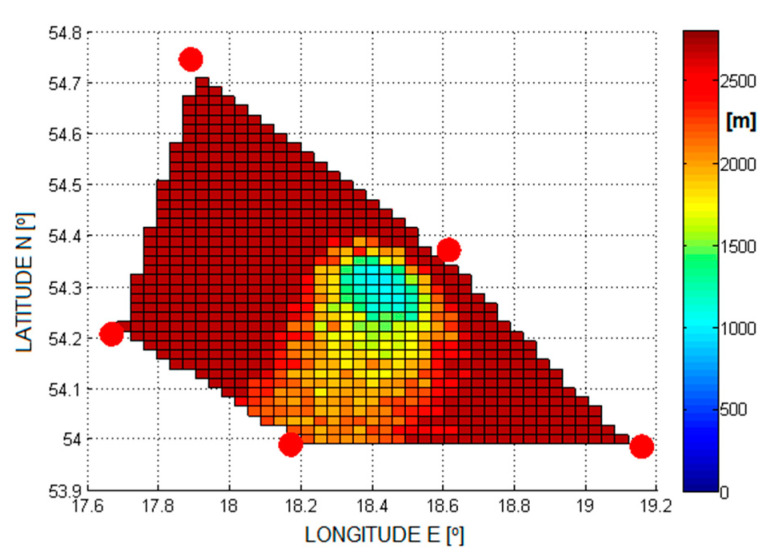
The RMSE of 2D position estimation in (m) for the SI algorithm (TDoA error is ±5 µs).

**Figure 8:**
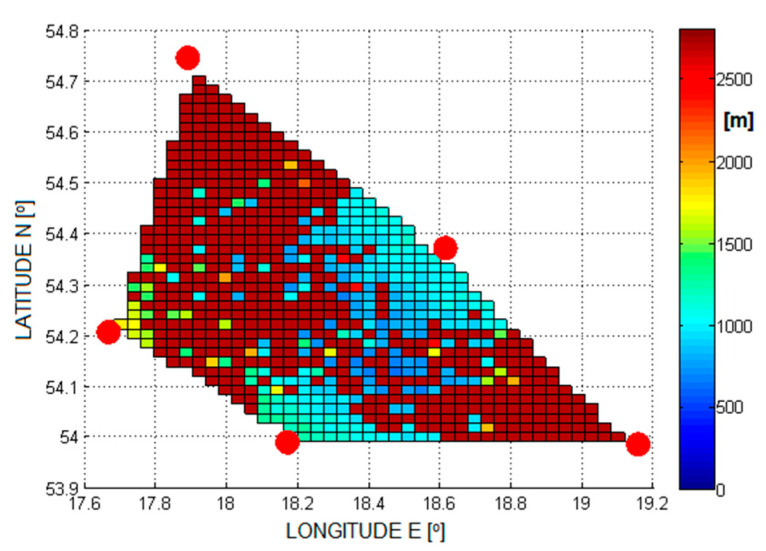
The RMSE of 2D position estimation in (m) for the Foy algorithm (TDoA error is ±5 µs).

Examples of the errors in the estimation of the aircraft position are shown in Figure 5, Figure 6, Figure 7 and Figure 8. Results for TDOA errors generated in range ±2 μs to ±5 μs were selected for presentation as we found from real field measurements that better quality of time measurements was very rare.

For the accepted TDoA errors, the best results were obtained for the Foy algorithm. Therefore, this algorithm became the leading algorithm during the real verification of the obtained results with the use of the technology demonstrator.

The quality of aircraft position estimation will not be the same in various parts of the considered airspace. To estimate the influence of aircraft position on its positioning accuracy, the concept of Position Dilution of Precision (PDoP) may be used [24,25,26,27]. In our case, the PDoP parameter is a measure of the impact of measurement errors on the estimated aircraft position [28]. The higher the value of this parameter, the lower the accuracy of the position estimation of the aircraft due to variable propagation of measurement errors in nonlinear positioning equations [29]. For the TDoA method in our system, PDoP is defined as the quotient of the aircraft position estimation error and the error of estimation of differences in distance between tracked transmitter and reference receivers on the ground. The PDoP coefficient can be determined based on the Jacobi matrix, which consists of the first-order partial derivatives of linearized positional equations:

(18) J⋅Δx=Δd

where **J** is the Jacobi matrix, Δ**x** is a vector of coordinates of the tracked aircraft, and Δ**d** is a distance difference vector. The PDoP coefficient can then be evaluated using the relationship [30]

(19) $$P_{D}=\sqrt{tr\left[{\left(J^{T}J\right)}^{-1}\right]}$$

where *tr*[·] is the trace of the matrix.

**Figure 9:**
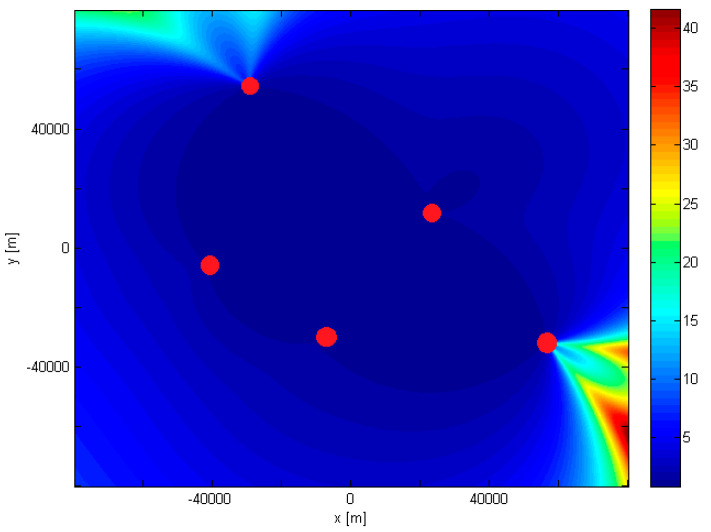
Accuracy fields (PDoP) of MLAT consisting of five stations.

Using (19), numerical calculations were carried out to determine the average value of the *P_D_* in the TDoA method in the area adopted for the study. Figure 9 shows the results, with the coordinates in the drawing reduced to the Universal Transverse Mercator (UTM) system concerning the center of the area defined by the coordinates of radio stations.

In an area bounded by radio stations, the average *P_D_* value does not exceed 3–4. Outside this area, the value of this coefficient is mostly within the range of 15. Figure 9 also shows a few areas where the value of this coefficient is within the range of 15 to 43. Based on this analysis, it can be concluded that the proposed locations of radio stations adopted for simulation and measurement tests are appropriate, because they ensure the lowest possible average value of the *P_D_* coefficient.

## 5. Experimental Research

In order for airplanes to be located with acceptable accuracy, it is necessary to develop the method of obtaining a sufficiently accurate TDoA estimate. In radiolocation systems, common practice is to determine the relative delay of signals by determining their cross-correlation function. However, it should be borne in mind that transmitters in these systems emit signals, the form of which is known on the receiving side. In addition, the waveforms of these signals are designed in such a way that they have good correlation properties giving a clearly marked unambiguous maximum suitable for time measurements. In the described VCS system, analog speech signals are used. These signals are generally of variable waveform so their correlation properties are also variable and what is more: the quality of correlation measurements of such signals depends on many factors, such as transmitted messages and voice characteristics of the speaking person. These signals are generally of unknown waveform and have a narrowband character different from noise. For such signals, an additional processing step is required to obtain a clear maximum of the correlation function.

Another issue concerning TDoA determination that needed to be solved was the limited time resolution of the correlation function. The signals received at the reference stations are sampled at a frequency of 40 kHz, which corresponds to a time interval of successive samples of 25 μs. In this case, TDoA time resolution would enable the determination of differences in the distance of the object from a pair of reference stations with a resolution of approximately 7500 m. Such a low resolution does not allow for determining the position of the aircraft with a sufficiently small error. To increase the time resolution of TDoA measurements, interpolation of the correlation function was used.

**Figure 10:**
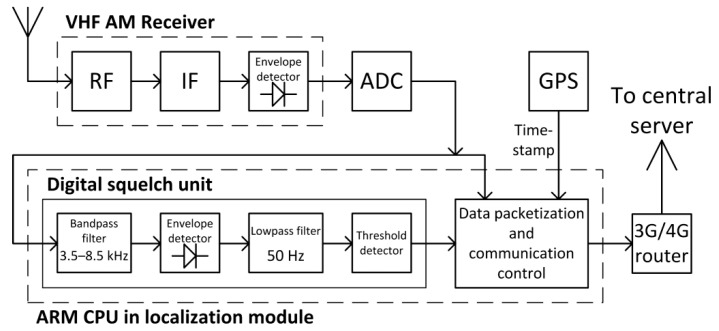
Block diagrams of signal processing in the radio station.

**Figure 11:**
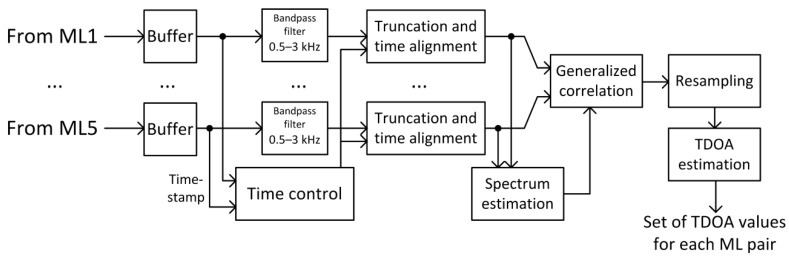
Block diagrams of signal processing in the central server.

Bearing in mind the above limitations, in order to implement a radiolocation system based on the analysis of received speech signals, the following block diagrams of signal processing in each radio station (Figure 10) and in the central server (Figure 11) were proposed.

On the radio station side, the received speech signals after AM demodulation in the envelope detector inside the air-band receiver are fed to a 14-bit analog-to-digital converter (Figure 10). With a predetermined sampling rate of 40 kSa/s, the samples of speech signals are then fed to the digital squelch unit, in which the following digital signal processing operations are performed in turn: bandpass filtering, envelope detection, lowpass filtering, and threshold detection. Samples of speech signals, for which the signal at the output of lowpass filter in squelch unit was below a predefined threshold, are then subject to packetization. Additionally, each packet is provided with control signals and timestamps from the GPS receiver. Measurement data is transmitted to the central server via a 3G/4G wireless router.

In turn, in the central server, the process of digital signal processing is carried out according to the diagram in Figure 11. Samples of speech signals preprocessed in radio stations are first buffered and then subjected to bandpass filtration. The next step in the processing in the central server is to determine the common part in the time domain of all sets of speech signal samples received by each radio station. In the final stage of signal processing on the central server, a transition to the frequency domain (spectrum estimation) takes place and generalized correlation is determined, resampling to ensure appropriate measurement resolution and TDoA time estimation for all pairs of radio stations.

Selected digital signal processing modules in radio stations and in the central server are described in detail later in this section.

### 5.1. Preprocessing of Speech Signals

Before calculating the correlation function of the signals received by the two reference stations, preprocessing of these signals is performed to eliminate components other than those associated with the transmission of speech signals.

First, noise segments at the beginning and end of the file containing the audio samples are detected and removed. The noise segments occur at the beginning and end of the actual voice message and they are results of both transient effects in onboard transmitter—this component should be correlated in all ground radio stations and transient effects in receivers which are not correlated nor synchronized between radio stations and must be removed. They can also appear in pauses between statements, but these segments are not removed.

**Figure 12:**
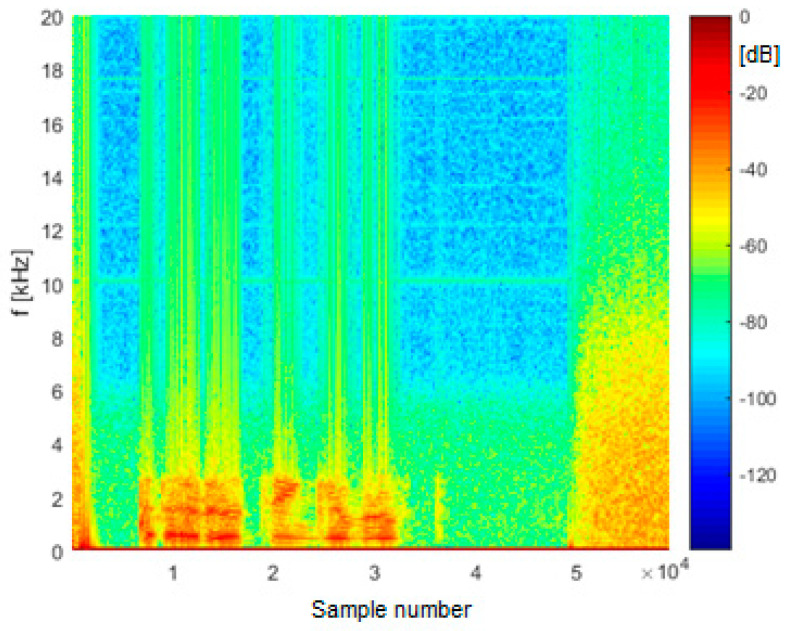
An example spectrogram of an audio file.

Spectrogram analysis shows that a major part of the speech signal power during air-to-ground communication is concentrated in the frequency band up to 3–3.5 kHz, which is mainly limited by characteristics of a human voice. On the other hand, in segments containing noise, the bandwidth goes up to 8–8.5 kHz, which is limited by the bandwidth of IF filters in the receiver. An exemplary spectrogram is shown in Figure 12.

**Figure 13:**
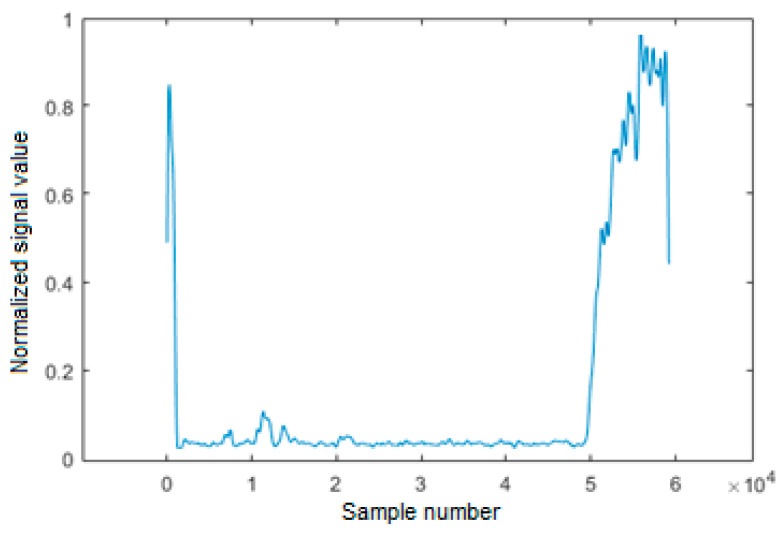
An example of the signal from envelope detector in squelch unit after smoothing and normalization.

Hence, the detection of noise segments is based on an analysis of the signal strength in the 3.5 kHz to 8.5 kHz band. First, the signal is filtered with a bandpass filter with such a passband. It is a filter with a finite impulse response, and its order is 1000. Another element of digital signal processing is the envelope detector. Thereafter, filtering of the signal is performed by a lowpass filter of 1000, with a cutoff frequency of 50 Hz. At the output of this filter, we have a signal which may be understood as short-term (tens of milliseconds) amplitude of noise component in samples of signal from AM receiver. The signal formed in this way is then normalized to the maximum value in a time window of 300 s to achieve automatic adaptation of detection threshold in case of variable noise level caused by industrial interferences. An example of the noise signal form after smoothing and normalization is shown in Figure 13.

It was assumed that noise occurs in those file segments where the value of the metric shown in Figure 13 exceeds the level of 0.3. Thus, noise at the beginning of the file occurs from the beginning of the file until it is below the threshold. In turn, at the end of the file, the noise covers its last segment where the value does not fall below the threshold. In addition, a margin of 1500 samples on each side is also removed to get rid of any transient effect which may occur at the beginning of signal reception (for example, distortion caused by the different behavior of automatic gain control in VHF AM receivers in different stations) and at the end (noise recorded after the end of transmission before squelch unit switched off signal recording). However, taking into account both operations, in each audio file, a different number of initial samples may be removed. Therefore, before computing the correlation function, timestamps are updated to be the same as the sampling time that occurs first in the file.

In the next preprocessing step, the waveform from the audio file, after cutting the starting and ending noise fragments, is filtered with a filter with a passband from 500 Hz to 3 kHz. This is to eliminate distortion outside the speech signal band. A filter with a finite impulse response, order 1000, is used.

### 5.2. Correlation of Speech Signals

Signals from VHF aviation radio are recorded in reference stations starting from the moment of their envelope detection. Due to the different strengths of the signals reaching the stations, the moment of their detection, and thus the start of recording, may differ. Nevertheless, information on the start of recording of each signal is available, relative to a common clock synchronized with the GPS receivers. The moment of starting the recording is determined with an accuracy of 1 μs.

Before determining the correlation function, the waveforms of the signals are aligned on the time axis. In the next step, circular correlation is determined, consisting of cyclically shifting one of the signals by one sample to find the shift for which the correlation reaches its maximum, i.e., for which the signals are closest to each other. Cyclic correlation requires that the signals have the same number of samples. In case one of them is shorter, it is padded at the end with zeros (zero padding).

The cyclic correlation is computed in the frequency domain using the fast Fourier transform (FFT). It is a method that has less computational complexity than computing time-domain correlations. The FFT correlation is based on the calculation of the cross-spectrum of two signals, i.e., the product of the spectrum of one signal and the complex conjugation of the spectrum of the other signal, then computing the inverse fast Fourier transform (IFFT) and determining the modulus from the result.

As mentioned, speech signals are not of a noise nature, and therefore there may not be one clearly marked maximum in the function of their correlation. Therefore, in the case of such signals, the generalized cross correlation is used [31]. It involves deliberate, preliminary shaping of the signals before calculating the correlation. It corresponds to passing the signals through a filter with a frequency response inverse to the signal spectrum, resulting in flattening the spectrum and thus imparting noise characteristics to the signal.

**Figure 14:**
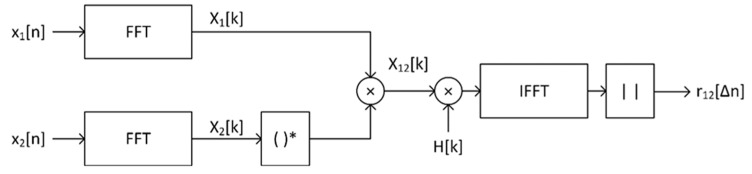
Scheme for computing a generalized cross-correlation function.

Figure 14 shows a diagram of the procedure for determining the generalized cross-correlation function with the use of the fast Fourier transform.

The cross spectrum of *x*_1_[*n*] and *x*_2_[*n*] is determined as

(20) $$X_{12}\left[k\right]=X_{1}\left[k\right]\cdot X_{2}^{*}\left[k\right]=FFT\left\{x_{1}\left[n\right]\right\}\cdot FFT{\left\{x_{2}\left[n\right]\right\}}^{*}$$

and generalized correlation

(21) $$r_{12}\left[\Delta n\right]=\left|IFFT\left\{H\left[k\right]\cdot X_{12}\left[k\right]\right\}\right|$$

where *H*[*k*] is the frequency response of the shaping filter.

Several variants of filtration are described in the literature, e.g., Roth, PHAT, and SCOT [23,31]. The last of them was chosen for implementation in the VCS system because it shows the most clearly marked maximum correlation. Its frequency response is determined as follows

(22) $$H_{SCOT}\left[k\right]=\frac{1}{\sqrt{X_{11}\left[k\right]\cdot X_{22}\left[k\right]}}$$

where *X_ii_*[*k*] is the auto spectrum of the *i*-th signal, determined analogously as in (20).

It has been observed that the generalized correlation function may show a false global maximum for ∆*n* = 0 in the case where the denominator values of the filter frequency response are close to zero. To prevent this, the lowest denominator value *H*[*k*] is increased to the level equal to 1% of the maximum value of this denominator.

**Figure 15:**
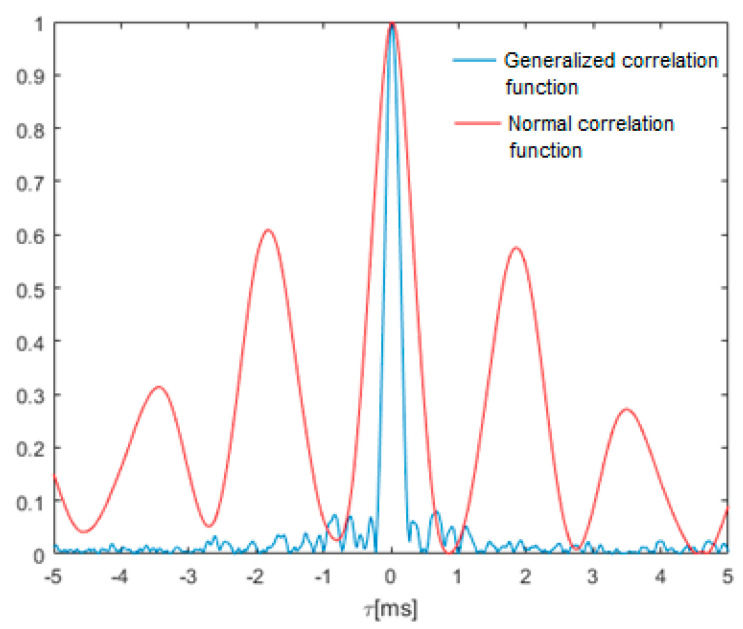
Comparison of the waveforms of the normal and generalized correlation function of the audio signal from an aviation radio.

Figure 15 shows a comparison of the normal and generalized correlation functions in the vicinity of the maximum. As can be seen, in the case of speech signals, there are several local maxima in the standard correlation function, and the peak width around the global maximum is significantly greater than in the case of the generalized correlation.

### 5.3. Interpolation of the Correlation Function

Increasing the accuracy of TDoA estimation requires increasing the time resolution of signal analysis. An interpolation method is used to estimate the sample values between the original samples of band-limited signals.

Two variants are possible—interpolation of received signals in the time domain or interpolation of the correlation function. The first of these increases the processing effort due to the need to calculate the long FFT transforms. Hence, the second option is preferred, as it enables us to obtain similar results with much lower computational complexity. Additionally, in this case the processing effort is limited because only a fragment of the correlation function is analyzed in the vicinity of its global maximum—there is no need to interpolate the entire correlation function. A segment of ±30 samples from the maximum is assumed to be interpolated.

The first step in the interpolation process is oversampling. A fixed number of zeros samples (*N_zer_*) are inserted after each original sample. This number depends on the expected increase in time resolution. For example, a tenfold increase in resolution requires nine zeros.

The next stage of interpolation is the filtering of the oversampled waveform. The interpolated signal is obtained at the output of the low-pass filter with a cut-off frequency of 5 kHz.

Obtaining an appropriately selective filtration requires the proper selection of the filter length with a finite impulse response. The empirically selected length is 400·(*N_zer_* + 1). A 100-fold oversampling of the correlation function was adopted; therefore, a FIR filter of 40,000 was used.

Zero-valued vectors with a length equal to one-half of the filter length are added at the beginning and end to the segment of the correlation function before filtering. In turn, many zeros are added to the impulse response of the filter so that it has the same number of samples as the waveform to be filtered. The filtration process itself is performed in the frequency domain using the FFT transform. After filtration, excess samples at the start and end of the waveform are removed to restore the original length of the oversampled correlation segment.

**Figure 16:**
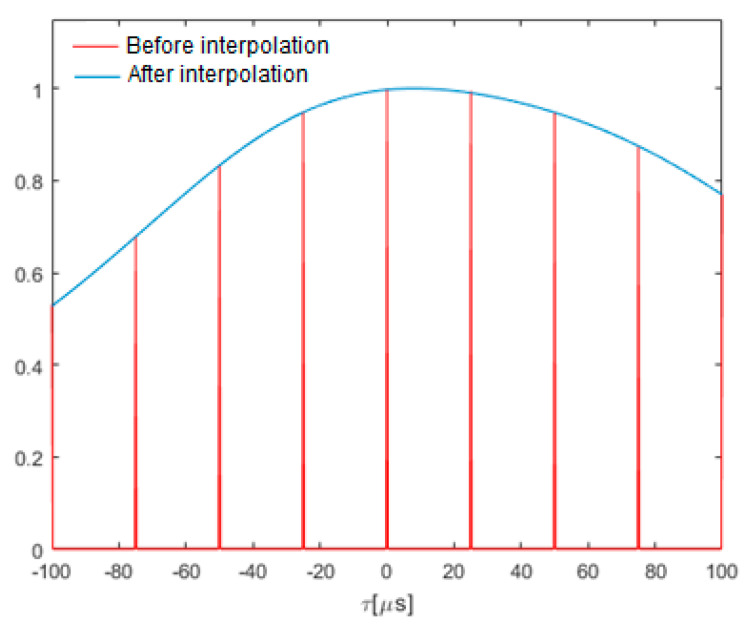
Comparison of the generalized correlation function before and after interpolation.

Figure 16 shows an example of the correlation function in the vicinity of the maximum. Red represents the oversampled waveform and blue represents the same waveform after lowpass filtration. There is a noticeable asymmetry of the correlation function around the maximum. In such a case, the maximum of the function after interpolation is closer to the actual delay between the analyzed signals—an increase in time accuracy is obtained.

The final value of the TDoA estimate is the sum of two components. The first is the difference of the timestamps associated with the times when the first samples of the correlated waveforms were taken. The second component is the shift of the signals corresponding to the maximum of the correlation function after interpolation.

### 5.4. Results of Measurement Tests

The first step in the aircraft positioning process is selecting a set of recorded signals for the same audio transmission received by different radio (reference) stations (RS). The grouping of signals is based on the comparison of the timestamps of their recording start (these markers are saved in the database).

The set includes signals that markers differ by no more than a preset time interval. This interval is defined by the operator and is one of the configuration parameters of the TDoA determination program.

Originally, the value of the maximum interval between the timestamps was set to 2 s. However, problems with incorrect grouping of data were observed with this setting, especially in the case of series of short voice messages lasting 1–2 s only. TDoA values derived from such a data set quite often exceeded one second, indicating that the signals could not be related to the same transmission. For this reason, it was decided to reduce the allowable timestamp difference to 500 ms. At RSs, the recording start timestamp is set with a step of 40 ms, which corresponds to a segment of 1600 samples at a sampling rate of 40 kHz. Possible timestamp differences are therefore a multiple of this interval.

**Figure 17:**
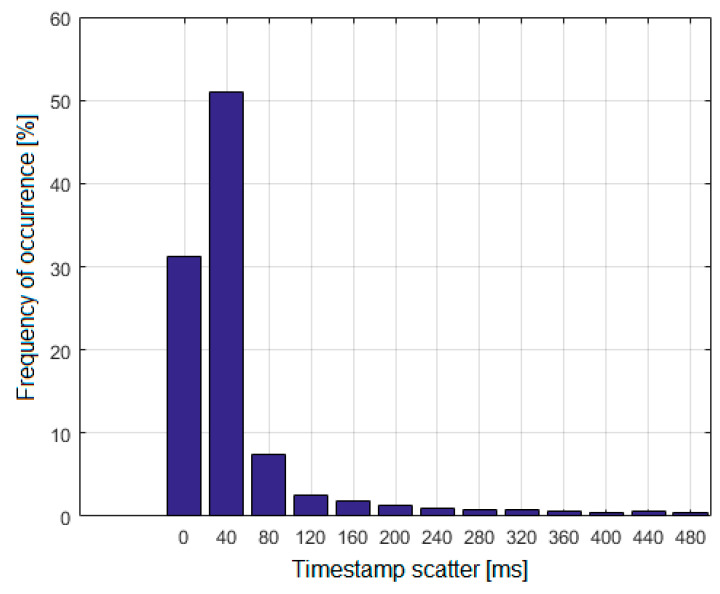
Scatter distribution of timestamps in voice data sets.

For each set of voice data, a time difference between the latest and the earliest marker is determined. The distribution of these values is presented in Figure 17.

The distribution was based on 6180 sets of voice data. As shown in Figure 17, over 80% of cases are the scatter values of 0 ms and 40 ms, and almost 90% of the values do not exceed 80 ms. It can be seen that relatively small scatter is the most common, which indicates that the voice data is grouped correctly. Grouped records were used to estimate TDoA values, but we limited possible length of voice signals to a range between 2 and 6 s (80 to 240 thousand samples). Messages shorter than 2 s were rejected because they resulted in large width of main correlation peak due to limited signal-to-noise ratio after correlation. On the other side, messages longer than 6 s were rejected because commercial planes, in 6 s, can travel more than 1.5 km which significantly changes time delay between reception of beginning and end of transmission in ground stations; therefore, main correlation peak tends to increase width.

Tests for determining the TDoA value were performed in two stages. The first stage concerned the evaluation of the TDoA scatter of the signal emitted by the ground transmitter (ATC ground transmitter for ground-to-air communication in the approach phase at the airport in Gdansk) and received by a pair of RSs no. 3 and no. 5. Data from one selected observation day were analyzed. The transmissions from the ground station were identified through direct listening—52 messages were identified. Due to the unknown location of the transmitting station, it was not possible to determine the actual TDoA value; however, approximately constant values were to be expected.

In the first approach, TDoA values of signals from ground ATC transmitter were calculated using standard correlation in time domain. Obtained results were oscillating around 190 μs, but they had a large scatter—the minimum value was 168 μs, and the maximum was 203 μs. The standard deviation was 5.3 μs. A few times smaller scatter was expected; therefore, modifications were made at the software code level for TDoA determination. These modifications included:

- Introduction of filtration to eliminate disturbances outside the speech signal band;
- Adding detection and elimination of the beginning and end of the transmission, containing noise related to switching on and off the transmission;
- Generalized correlation estimated in frequency domain instead of standard correlation in time domain.

**Figure 18:**
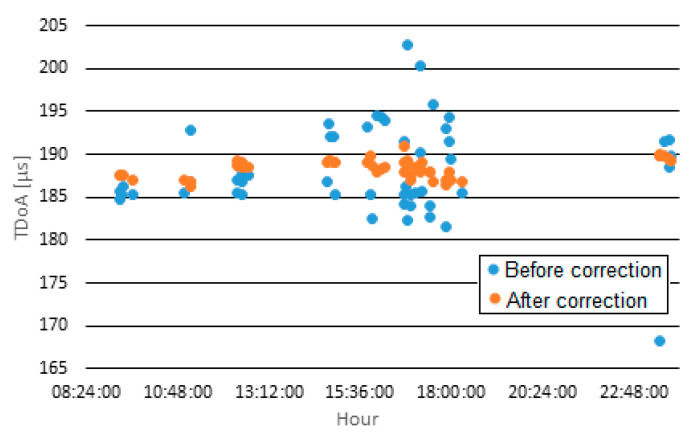
TDoA values from the ground transmitter recorded by radio stations no. 3 and no. 5.

After making the above changes, the scatter of estimated TDoA values decreased significantly. The variability of these values is shown in Figure 18. This time, a standard deviation of approximately 1 µs was obtained, and the difference between the maximum and minimum TDoA values did not exceed 5 µs. These are the values to be expected with the good quality transmission of signals received by radio stations.

The second stage of verifying the correctness of TDoA determination was to compare the TDoA values calculated by the server with the values corresponding to the actual positions of the aircraft. Information on reference (actual) position was obtained from the ADS–B system data stored in the central server database.

Data from nearly one month were analyzed. Unfortunately, these tests were conducted after COVID lockdown so the number of flights in the supervised area was significantly reduced and so the amount of data for system performance evaluation was also limited. First, sets of TDoA values related to the same voice transmission were mapped to the ADS–B data as follows:

- Based on the position from the ADS–B system for a given second (the same as the TDoA timestamp) and the coordinates of the position of the RS, the reference TDoA values were calculated;
- Then the ADS–B position for which the estimated TDoA values were most similar to the reference ones was selected;
- An aircraft callsign associated with the selected ADS–B position was searched in the database.

The initial linkage of the TDoA data to ADS–B was further verified by determining whether the aircraft callsign provided in the voice message matched that contained in the ADS–B data. TDoA and ADS–B data set pairs where the callsign was not compliant or could not be verified were rejected. For the remaining data, the RMSE for TDoA was determined

(23) $$RMSE_{TDoA}=\sqrt{\frac{1}{N}\sum_{n=1}^{N}{\left(TDoA_{est,n}-TDoA_{ADS-B,n}\right)}^{2}}$$

where *N* is the number of the radio station pair between which TDoA is determined, *TDoA_est_* is the value estimated by the central server, and *TDoA_ADS-B_* is the value determined based on the position from ADS–B.

**Figure 19:**
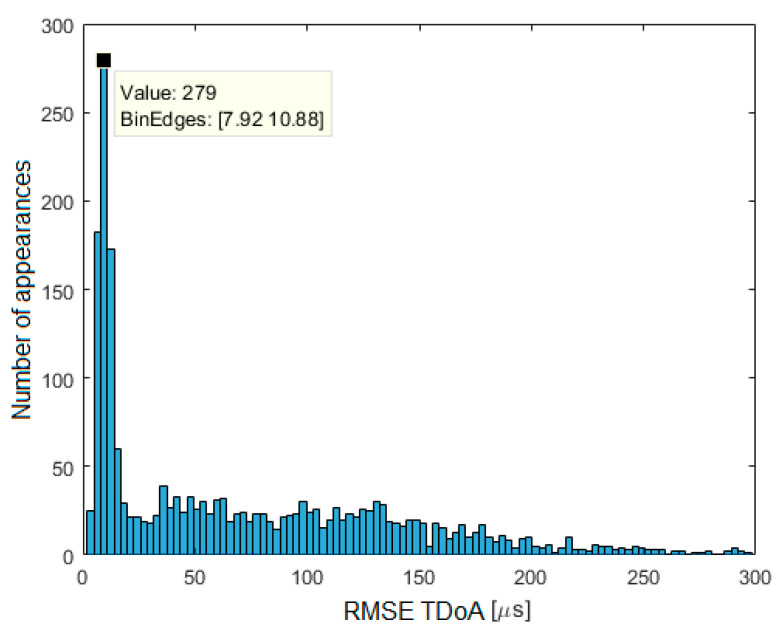
Distribution of TDoA estimation error from collected data over a one-month period.

The TDoA error histogram is presented in Figure 19. It was determined on the basis of 2078 pairs of TDoA–ADS–B data sets, where the RMSE did not exceed 300 μs. As may be seen, the most common RMSE TDoA is between 8 μs and 11 μs. Based on this data, the mean TDoA error in each pair of RSs was calculated. This was to determine whether the TDoA error was systematic and whether it could be compensated by adding corrections to the timestamps. The presence of TDoA errors limited to a few microseconds is fully justified by the different delays of signals in filters inside VHF AM receivers, especially 2nd IF filters. These systematic time measurement errors may be compensated by system calibration.

Table 2 gives the values of the mean error of TDoA estimation. Ideally, the error determined for the station pair *M,N* should be the opposite of the error for the station pair *N,M*. Results presented in Table 2 were grouped depending on which radio station assigned earlier timestamp to the recorded stream of samples.

Those cells are marked in green where the discrepancy between errors, after changing the order of stations, in terms of the absolute value, does not exceed 1 μs. Yellow indicates a discrepancy of 1 to 2 μs, orange—between 2 and 3 μs—and red—more than 3 μs. As can be seen, in the case of 7 out of 10 pairs of reference stations, there is a clear error symmetry (the discrepancy does not exceed 2 μs). This indicates that the TDoA error may be systematic and it is desirable to try to compensate for it.

To determine the timestamp corrections for individual RS, based on the values in Table 2, a system of linear equations was created in the following matrix form **Ax** = **b**

(24) $${\left[\begin{array}{ccccc} 1 & -1 & 0 & 0 & 0\\ 1 & 0 & -1 & 0 & 0\\ \vdots & \vdots & \vdots & \vdots & \vdots \\ 0 & 0 & -1 & 0 & 1\\ 0 & 0 & 0 & -1 & 1 \end{array}\right]}_{20x5}\cdot {\left[\begin{array}{c} x_{1}\\ x_{2}\\ x_{3}\\ x_{4}\\ x_{5} \end{array}\right]}_{5x1}={\left[\begin{array}{c} 10.7487\\ 10.0066\\ \vdots \\ 4.6863\\ -5.1527 \end{array}\right]}_{20x1}$$

In matrix **A**, the value 1 is entered in the column corresponding to the reference radio station index and the value -1 in the column corresponding to the index of the second RS station in the pair. The rest of the matrix is zero. The elements of the vector **x** are the desired timestamp corrections for RS from no. 1 to no. 5 and vector b is created from corresponding TDoA error values from Table 2. The system of equations has been solved by the least squares method

(25) $$\hat{x}=A^{+}b$$

where **A**^+^ is a pseudo-inverse matrix to **A** (pinv() function in MATLAB environment). The correction values determined in this way are included in Table 3.

The accuracy of aircraft position estimation by the technology demonstrator was determined by comparing it with the positions reported in the ADS–B system messages transmitted from ADS–B equipment onboard aircraft. Information about the current position of the aircraft in ADS–B messages comes from the onboard GPS receiver. Thus, the accuracy of the aircraft position used as reference data in our investigation is determined by the class of the GPS receiver used in the aircraft, method of data processing in ADS–B transmitter, and probably also delay between position estimation and message transmission. It results from [32,33] that the accuracy of the position reported by the ADS–B system in the horizontal plane cannot be worse than 0.05 nautical mile, that is approximately 90 m. Thus, it is at least an order of magnitude better than the tested VCS-MLAT system.

The basic analysis performed was the estimation of the error in the horizontal plane related to the longitude and latitude of the localized object. The results for the two position estimation algorithms, Foy and SI, were obtained based on data from the two-month observation period.

**Figure 20:**
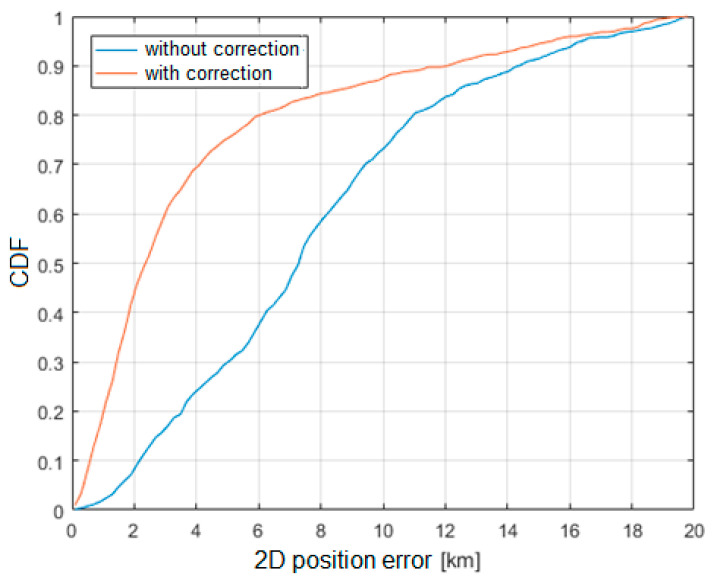
Cumulative distribution function of horizontal position estimation error (Foy algorithm).

**Figure 21:**
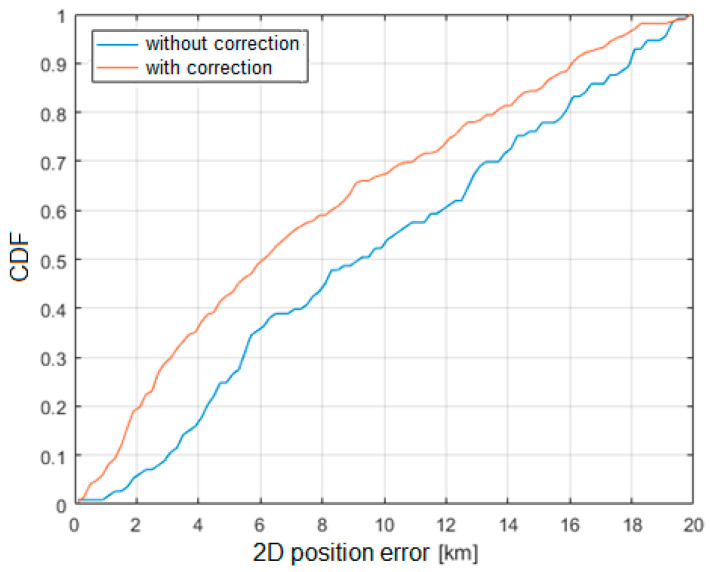
Cumulative distribution function of horizontal position estimation error (SI algorithm).

Figure 20 and Figure 21 show the cumulative distribution function (CDF) of the 2D position estimation error (horizontally) for the two above-mentioned algorithms. There are two curves in each figure—the red one indicates the result after applying timestamp corrections, while the blue line is for the case without the use of time corrections. It can be seen from these figures that corrected results of position estimation are worse than results of simulations taken with assumption that TDOA errors are limited to ±2 μs (Figure 5 and Figure 6). However, simulation results obtained for TDOA errors in range ±5 μs (Figure 7 and Figure 8) are in quite good agreement with cumulative distribution charts of position estimation error from real field measurements.

It should be added that the results of aircraft position measurements obtained from the system tests in real conditions did not undergo any additional processing. The inability to automatically identify the aircraft on the basis of the recorded voice signals does not allow combining the results of subsequent measurements and applying averaging or filtering the position; therefore, each result of position estimation must be treated separately.

The results clearly indicate that the SI algorithm shows a significantly greater error in the position estimation compared to the Foy algorithm. This effect may be caused by the non-Gaussian distribution of errors in measured TDoA values and possibly correlation between measurement errors while least-squares algorithms expect input data to have uncorrelated Gaussian error characteristics. Additional degradation in position calculation quality may occur with an overdetermined set of equations where signals are received simultaneously by more than three ground receivers. Inconsistent measurement errors, in this case, emphasize the differences between different position calculation algorithms. It is also worth mentioning that TDoA measurements were made in a 3D environment, and the position estimation was limited to the 2D case only. In this case, a nonlinear transformation of the error distribution takes place, which may affect the positioning algorithms in different ways. Future work may include estimating the error distribution and optimizing the position computation algorithm for voice-based multilateration.

**Figure 22:**
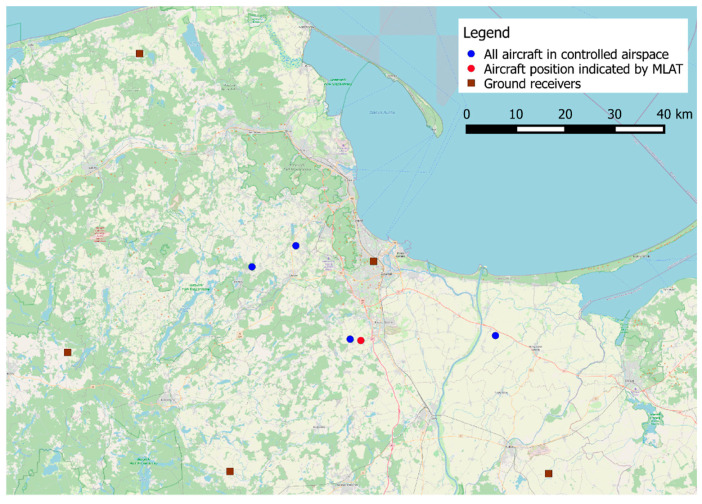
Exemplary map with single result of aircraft position estimation using voice transmission in VHF band.

Although presentation of position estimation results on a map may provide useful information, e.g., on distribution of errors and systematic shifts, we found that plotting over 6000 processed records on one map makes it unreadable. Instead, we present in Figure 22 one example of aircraft position estimation using recorded voice communication in VHF band (red dot) together with true coordinates from the ADS–B system of all aircraft which were present at the same time in controlled airspace around Gdansk airport (blue dots). In this example the position estimation error was equal to 3.7 km. However, even such large error is smaller than minimal aircraft separation defined for this part of airspace (5 km) and it is clearly visible from the map which aircraft transmitted voice messages. Therefore, our solution may be used for quick verification of transmitting aircraft even when pilots skip aircraft callsigns in their transmission.

## 6. Conclusions

This paper describes in detail the innovative method of locating airplanes based on audio signals received by several radio stations deployed over a given area. Audio signals are emitted in the 118–136 MHz band by on-board radio stations during voice calls of aircraft pilots with the air traffic control tower. The developed method can be successfully used as an extension of the voice communication system (VCS) standard, which is currently being developed in the world, which is one of the important elements of air traffic management (ATM).

To verify the developed method, the technology demonstrator was made, in which five radio stations (RS) distant from the airport by approximately 60 km were located in a given area of northern Poland. The connections of the radio stations with the central server, on which the software for estimating the position of the aircraft was installed, were made via the cellular network. In the adopted implementation of radio stations that use the public LTE network for communication with the system server, the emphasis was placed on the reliability and credibility of data transmission, so the packet transmission mode was used without guaranteed low delays. Multiple queuing of transmitted speech signals, first in localization modules (LM), then in LTE routers (UMAD) and on the system server in order to estimate and compensate for delays, makes such a combined audio signal a delayed signal from single seconds up to several dozens of seconds. In real implementations, the backbone of the VSC system should be based on fixed networks (e.g., fiber optic). Another inconvenience in estimating the accuracy of the aircraft position estimation was difficult to predict shifting of the timestamps of the collected audio files with the model aircraft position obtained from the ADS–B system. In the developed localization system, there is no correlation between audio signals and data generated independently from the ADS–B system.

The main result obtained from the conducted experimental studies is the cumulative distribution function (CDF) of the error of the airplane position estimation. Based on the obtained results, it can be concluded that in approximately 50% of all the considered cases, the error in estimating the position of the aircraft did not exceed 2000 m, which, assuming the speed of the localized aircraft at 900 km/h (250 m/s), should be considered satisfactory and acceptable in practical applications. The transition from the demonstration phase to the implementation of the presented method will certainly make it possible to increase the accuracy of the aircraft position estimation after eliminating the shortcomings described in the paper.

Despite the above-mentioned disadvantages, it is worth noting that the developed innovative method of locating aircraft based on speech signals can be used as an alternative to the existing ones. At this point, there are many potential scenarios where the new method would be a valuable complement to the currently used aircraft navigation systems. The proposed solution may be used as a backup positioning system in case of primary systems failure, need for position estimation of aircraft not equipped with SSR or ADS–B devices, or as a tool for quick verification of transmitting aircraft, for example in the case of no callsign exchange. It is worth mentioning the cases of deliberately concealing one’s position in the airspace or failure of the existing radio navigation systems, and the only means of communication between the plane and the traffic controller is the AM radio.

## Figures and Tables

**Table 1:** Reference station positions in the simulation.

Station Number	Latitude N [°]	Longitude E [°]	Altitude above Sea Level [m]
1	54.37072	18.61375	43
2	54.20667	17.66889	191.3
3	53.99089	18.17064	100.0
4	54.74250	17.89111	74.6
5	53.98694	19.15492	24.0

**Table 2:** Average TDoA error (in microseconds) for individual pairs of radio stations. Cell background colors are explained in text below table.

		Radio Station Number				
		1	2	3	4	5
Radio station number	1	-	10.7487	10.0066	−1.8848	5.8604
	2	−9.9878	-	−2.9358	−12.5920	−5.8638
	3	−9.4959	−0.2772	-	−3.5793	−5.6333
	4	3.6242	12.3542	10.9874	-	7.2628
	5	−4.3767	5.64306	4.6863	−5.1527	-

**Table 3:** Assigned values of timestamp correction for radio stations.

Radio Station Index	Timestamp Correction [µs]
1	4.497
2	−5.985
3	−4.173
4	5.744
5	−0.083

## Data Availability

Data sharing not possible due to restrictions of Polish law (distributing confidential correspondence is prohibited).
